# Human papillomavirus type 16 infection activates the host serine arginine protein kinase 1 (SRPK1) – splicing factor axis

**DOI:** 10.1099/jgv.0.001402

**Published:** 2020-03-13

**Authors:** Sarah Mole, Arwa Ali A. Faizo, Hegel Hernandez-Lopez, Megan Griffiths, Andrew Stevenson, Sally Roberts, Sheila V Graham

**Affiliations:** ^1^​ MRC – University of Glasgow Centre for Virus Research, Institute of Infection, Immunity and Inflammation, College of Medical Veterinary and Life Sciences, University of Glasgow, Garscube Estate, Glasgow G61 1QH, UK; ^2^​ Institute of Cancer and Genomic Sciences, Institute of Biomedical Research West, College of Medical and Dental Sciences, University of Birmingham, Birmingham B15 2TT, UK; ^†^​ Present address: GlaxoSmithKline, Stevenage, UK; ^‡^​ Present address: Special Infectious Agents Unit, King Fahd Medical Research Center, King Abdulaziz University, Jeddah, Saudi Arabia; ^§^​ Present address: Bristol-Myers Squibb, Mexico City, USA

**Keywords:** human papillomavirus, infection, epithelial differentiation, tumour progression, cervical cancer

## Abstract

The infectious life cycle of human papillomaviruses (HPVs) is tightly linked to keratinocyte differentiation. Evidence suggests a sophisticated interplay between host gene regulation and virus replication. Alternative splicing is an essential process for host and viral gene expression, and is generally upregulated by serine arginine-rich splicing factors (SRSFs). SRSF activity can be positively or negatively controlled by cycles of phosphorylation/dephosphorylation. Here we show that HPV16 infection leads to accumulation of the paradigm SRSF protein, SRSF1, in the cytoplasm in a keratinocyte differentiation-specific manner. Moreover, HPV16 infection leads to increased levels of cytoplasmic and nuclear phosphorylated SRSF1. SR protein kinase 1 (SRPK1) phosphorylates SRSF1. Similar to HPV upregulation of SRSF1, we demonstrate HPV upregulation of SRPK1 via the viral E2 protein. SRPK1 depletion or drug inhibition of SRPK1 kinase activity resulted in reduced levels of SRSF1, suggesting that phosphorylation stabilizes the protein in differentiated HPV-infected keratinocytes. Together, these data indicate HPV infection stimulates the SRPK1–SRSF axis in keratinocytes.

## INTRODUCTION

Human papillomaviruses (HPVs) infect cutaneous and mucosal epithelia and cause mainly benign lesions (warts). However, a subset of HPVs which infect the anogenital epithelia can cause preneoplastic disease, which in rare cases can progress to cancers, most notably cervical cancer [1]. The most prevalent HPV worldwide is HPV type 16 (HPV16). HPV16 is responsible for 55 % of cases of cervical cancer but is also associated with over 30 % of oropharyngeal cancers, which is particularly prevalent in men [2]. Although much is known about the cancer-causing properties of HPV16, many aspects of the viral life cycle and its interaction with the host epithelial cell (keratinocyte) remain unclear. For example, our understanding of how the virus utilizes host gene expression mechanisms during its replicative life cycle remains incomplete.

HPV16 gene expression is stimulated or repressed at various post-transcriptional levels, including RNA splicing and polyadenylation [3, 4], mRNA stability, nuclear export and translation [5]. Cellular RNA processing factors including serine arginine-rich (SR) splicing factors (SRSFs) and heterogeneous ribonucleoproteins (hnRNPs) have been strongly implicated in HPV gene regulation. For example, SRSF1 and SRSF3 can stimulate, while hnRNP A1 and hnRNP H can repress, expression of the viral late mRNAs [4, 6]. Moreover, SRSF2, SRSF3 and hnRNP A1 can each activate alternative splicing of the early E6 and E7 mRNAs [7, 8]. SRSFs have multiple roles in RNA biogenesis, including in transcription elongation, splicing, nuclear export, mRNA stability and translation [9]. However, SRSFs are involved in many other cellular processes such as chromatin remodelling, genome stability and maintenance, nucleolar stress response, cell cycle progression and apoptosis control [10]. SRSFs allow appropriate gene expression in other viruses including herpes simplex virus type 1 (HSV1) [11] and human immunodeficiency virus (HIV) [12].

SRSF activity in splicing is determined by cycles of phosphorylation/dephosphorylation [13]. Phosphorylation is required for nuclear import of SR proteins [14, 15] while dephosphorylated SR proteins can accumulate in the cytoplasm due to their interaction with mature mRNAs exported from the nucleus [16–18]. SR protein phosphorylation is carried out *in vivo* by cyclin-dependent-like kinases (Clks) and SR protein-specific kinases (SRPKs) [13]. While Clks are found exclusively in the nucleus, SRPKs are present in both nuclear and cytoplasmic compartments. SRPK1 can bind nuclear Clk1 to promote splicing [19] and can relocate to the nucleus upon cell stress, in response to epidermal growth factor (EGF) and Akt signalling, and during the cell cycle [20]. SRPKs are examples of moonlighting proteins with multiple functions [21], because they can phosphorylate a range of proteins that do not have roles in RNA processing, for example they control metabolic signalling [20] and innate immunity [22].

The SRPKs are important factors controlling replication of DNA and RNA viruses. For example, HSV1 ICP27 protein interacts with SRPK1, controls SRSF phosphorylation and inhibits HSV splicing [23]. SRPK1 and 2 phosphorylation of the hepatitis B virus (HBV) core protein is an essential step in viral DNA synthesis [24] and SRPKs are used by HBV as chaperones in genome packaging [25, 26]. SRPK1 has been shown to have antiviral properties through regulating innate immunity [22]. In the case of HPVs, several HPV E2 proteins (the viral transcription/replication factor) in particular HPV1 and HPV8 E2, can bind SRPK1 directly [27] but the functional consequence of binding is not clear. HPV1 E2 is a substrate of SRPK1, while HPV1 E4 can inhibit SRPK1 and thus inhibit phosphorylation of SR proteins and HPV E2 [28].

Here we report HPV16 upregulation of SRSF1 phosphorylation and HPV-associated changes in the subcellular location of SRSF1 and its kinase, SRPK1, in infected keratinocytes. SRPK1 levels were induced in HPV16-positive differentiated keratinocytes. HPV16 E2, which can bind and activate SRSF gene expression and splicing [29–31], can stimulate SRPK1 expression.

## Methods

### Cell lines, drug treatment, ectopic expression and siRNA knockdown

W12E (clone 20863) [32], NIKS [33] and NIKS16 (clone 2L) cells [34] were grown in F-medium [32] on mitomycin C-treated 3T3 fibroblast feeder layer cells at a seeding ratio of 1 : 5 fibroblasts to keratinocytes at a concentration of 2×10^5^ cells per 100 mm dish. Differentiation was induced by culturing to high colony density in the presence of 1.2 mM Ca^2+^ [32]. U2OS cells stably transfected with an empty vector (U2OSV) or with a plasmid expressing HPV16 E2 (U2OS clones A4, B1) [35, 36], HaCaT cells and HeLa cells were grown in Dulbecco's modified Eagle medium (DMEM) with 10 % FCS (Invitrogen).

Cells were treated with SRPIN340 (Sigma) dissolved in DMSO for the stated times. E4 expression plasmid pMV11 (gift of Prof. John Doorbar, University of Cambridge) was transfected into HeLa cells using Lipofectamine 2000 (Invitrogen) according to the manufacturer’s protocol. Protein expression was examined after 48 h. SRPK1 was small interfering RNA (siRNA)-depleted by transfecting Dharmacon SMART-Pool siRNAs in RNAiMax transfection reagent (Invitrogen) mixture into undifferentiated NIKS16 cells, and then allowing them to differentiate as previously described [30]. siGLO was used as a non-target siRNA control and to monitor transfection efficiency.

### Protein extract preparation and western blotting

Cells were washed twice in PBS at 4 °C and lysed in 2× BOLT protein loading buffer (Invitrogen). Protein extracts were syringe-passaged through a 22-gauge needle 15 times then sonicated in a Sonibath for three 30 s pulses. The samples were boiled at 100 °C for 5 min before loading on a 12 % NuPAGE gel (Invitrogen) and electrophoresed at 150 V for 1 h in 1× MES buffer. Proteins were transferred to a nitrocellulose membrane using the iBlot transfer kit and iBlot Gel Transfer Stacks (Invitrogen) as per the manufacturer’s instructions. Membranes were blocked in 5 % milk powder in PBST (or in 2 % BSA TBST for the E6 blot) at room temperature for 1 h. Membranes were washed three times in PBST (or TBST for phosphoproteins) for 5 min each then incubated with the following primary antibodies: SRSF1 1 : 1000 (Zymed Laboratories, clone 96), SRSF2 1 : 1000 (Abcam), SRPK1 1 : 500 (BD Transduction Laboratories, clone G211-637), α-tubulin 1 : 5000 (Abcam), involucrin 1 : 1000 (Sigma clone SY5), GAPDH 1 : 1000 (Biodesign clone 6C5) and HPV16 E2 antibody 1 : 500 (Santa Cruz TVG271). Monoclonal antibody 104 (Mab104), which detects phosphorylated SR proteins, was prepared from hybridoma supernatants (ATCC CRL-2067) and used neat. The blots were incubated in their respective antibody for 1 h at room temperature or overnight at 4 °C. After 1 h, the blots were washed three times in PBST or TBST for 5 min. They were then placed in secondary antibody for 1 h [HRP-linked goat anti-mouse or goat anti-rabbit (Pierce) were used at 1 : 2000 dilution]. Blots were washed three times in PBST for 5 min before being incubated with ECL western blot substrate. The blots were exposed to X-ray film (ThermoScientific) and processed in an X-Omat processor, or imaged using an Odyssey LiCOR CLx infrared imaging system.

### Phosphoprotein analysis

Cells were scraped into NP-40 lysis buffer (50 mM Hepes, pH 7.5, 50 mM NaCl, 0.1 % NP-40) with fresh protease and phosphatase inhibitor cocktails according to the manufacturer’s instructions (Roche). Dephosphorylation was carried out by incubation with calf intestinal alkaline phosphatase (Invitrogen) exactly as described previously [37].

### Immunofluorescence microscopy

Cells were grown on sterile coverslips until 90 % confluent, then washed three times with PBS. Cells were fixed in 58 mM sucrose/5 % formaldehyde in PBS for 10 min at room temperature, and permeabilized with 70 % acetone/30 % methanol for 5 min at −20 °C. Alternatively, for detection of nuclear proteins, permeabilization was carried out with 0.5 % NP-40 in PBS for 5 min at room temperature. Coverslips were washed three times in PBS and incubated at room temperature for 1 h with primary antibody in PBS/10 % FCS and then washed three times with PBS followed by a final wash with distilled H_2_O. Antibodies were: SRSF1, clone 96, 1 : 250 (Zymed Laboratories); SRSF2, 1 : 250 (Sigma); SRSF7, clone 98, 1 : 25 (kind gift of Dr James Stevenin, Strasbourg); SRPK1, 1 : 250 (BD Transduction Laboratories); involucrin, 1 : 1000 (Sigma); and HPVE4, clone B11, 1 : 300 (kind gift of Prof. John Doorbar, University of Cambridge). DAPI and secondary antibodies were diluted in blocking solution and added to the cells for 1 h protected from the light before six washes in PBS, followed by one wash in distilled H_2_O. Coverslips were mounted on glass slides with a glycerol-based mounting medium (Citifluor, AF1) and sealed with nail enamel. Samples were examined using a Zeiss LSM 710 confocal microscope, and Zen black software (Zeiss) was used for capturing images. Image fluorescence was quantified using ImageJ following conversion to 8-bit format. Background was subtracted and integrated pixel density was calculated. Statisical analysis was carried out using Graphpad Prism version 8 software.

## RESULTS

### SRSF1 subcellular location is controlled by HPV-infection

We used two models of the HPV16 infectious life cycle, W12 and NIKS16 keratinocytes. W12E (clone 20683) cells are cervical keratinocytes derived from a low-grade cervical lesion that contain ~100 episomal copies of the HPV16 genomes [32, 38]. NIKS cells are spontaneously immortalized foreskin keratinocytes [33], and NIKS16 cells (clone 2L) are NIKS cells stably transfected with episomal HPV16 genomes [33, 34]. These three cell lines can differentiate in monolayer culture to express markers of differentiation such as involucrin, filaggrin and keratin 10. W12E and NIKS16 also synthesize HPV late proteins E4 and L1, suggesting completion of the viral life cycle [30, 36].

**Fig. 1.**
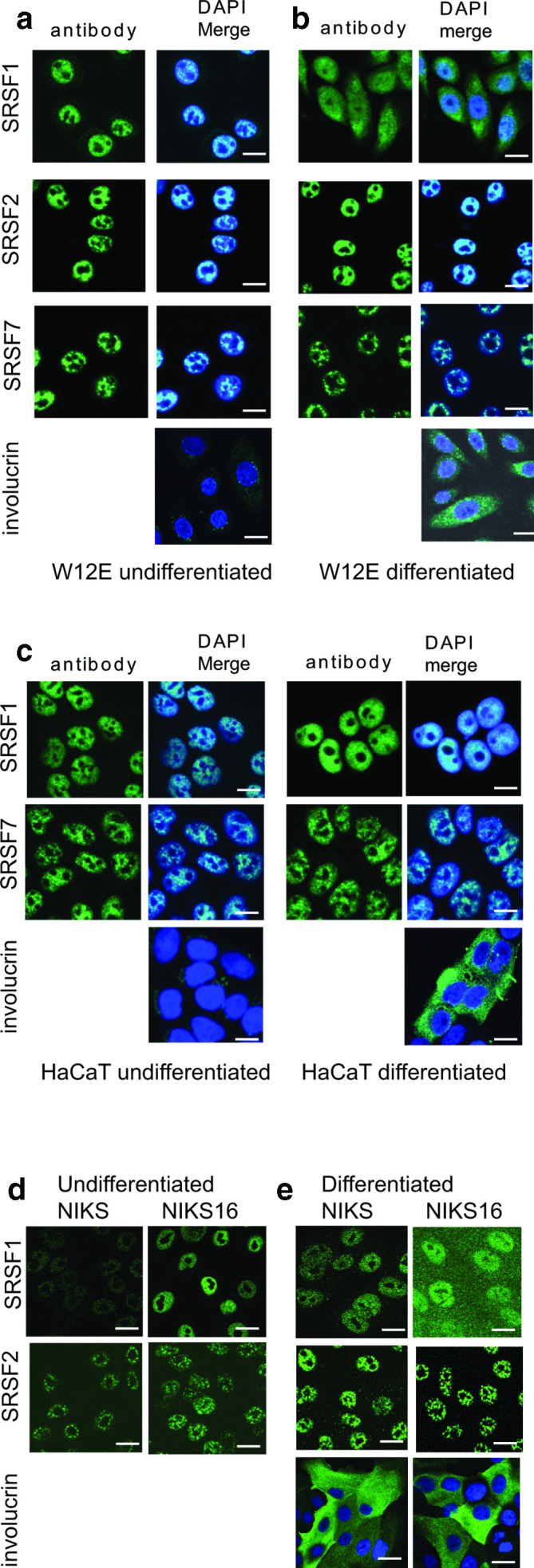
SRSF1 is present in the cytoplasm in differentiated HPV-infected keratinocytes. (a) Confocal microscopy analysis of the location of SR proteins SRSF1, SRSF2 and SFSR7 in undifferentiated W12E (HPV16-infected) keratinocytes. (b) Analysis of the location of SR proteins SRSF1, SRSF2 and SFSR7 in differentiated W12E (HPV16-infected) keratinocytes. (c) Analysis of the location of SRSF1 and SFSR7 in undifferentiated and differentiated HaCaT keratinocytes (HPV-negative). (d) Analysis of the location of SRSF1 and SRSF2 in undifferentiated NIKS (HPV-negative) and NIKS16 (HPV16-infected) keratinocytes. (e) Analysis of the location of SRSF1 and SRSF2 in differentiated NIKS (HPV-negative) and NIKS16 (HPV16-infected) keratinocytes. Cells were stained with involucrin to show differentiation. No involucrin staining was detected in undifferentiated cells. Nuclei were counterstained with DAPI (DAPI Merge). Bar, 20 µm.

SRSFs are transcriptionally upregulated at late stages of HPV infection [30, 36, 39, 40] and SRSFs have well-documented roles in positively and negatively controlling splicing of HPV RNAs encoding the viral early and late proteins [6, 8, 30, 41]. SRSFs can have cytoplasmic as well as nuclear functions [13, 42]. We examined the location of the HPV-upregulated SRSF protein SRSF1 in undifferentiated (early life cycle) and differentiated (late life cycle) HPV16-infected W12E cells. SRSF2 and SRSF7 were used as negative controls. Although SRSF2 is upregulated by HPV E2 [30], it is confined to the nucleus [43] while SRSF7 is not regulated by HPV16 [30]. SRSF1 was located mainly in the nucleus of undifferentiated W12E cells (Fig. 1a). In contrast, while SRSF2 and 7 remained in the nucleus when the cells were differentiated, SRSF1 was present in both the nucleus and the cytoplasm (Fig. 1b), indicating a differentiation-specific mechanism of relocation to the cytoplasm. Similar to SRSF7, SRSF1 was detected in the nucleus and was absent from the cytoplasm of HPV-negative undifferentiated and differentiated HaCaT keratinocytes (Fig. 1c). To determine if the relocation was due to HPV infection we compared SR protein location in NIKS and NIKS16 cells. SRSF1 and SRSF2 were located mainly in the nucleus of undifferentiated NIKS and NIKS16 cells (Fig. 1d). While SRSF2 was detected mainly in the nucleus of both cell types, SRSF1 was found in the cytoplasm of differentiated NIKS16 (although cytoplasmic relocation was not as striking as in W12E cells) but not in the HPV-negative NIKS cells (Fig. 1e). Involucrin levels were detected only in differentiated cell populations as expected. Together, these data suggest that HPV16 infection causes SRSF1 protein to accumulate in the cytoplasm of differentiated keratinocytes.

### SRSF1 phosphorylation is altered by HPV infection

**Fig. 2.**
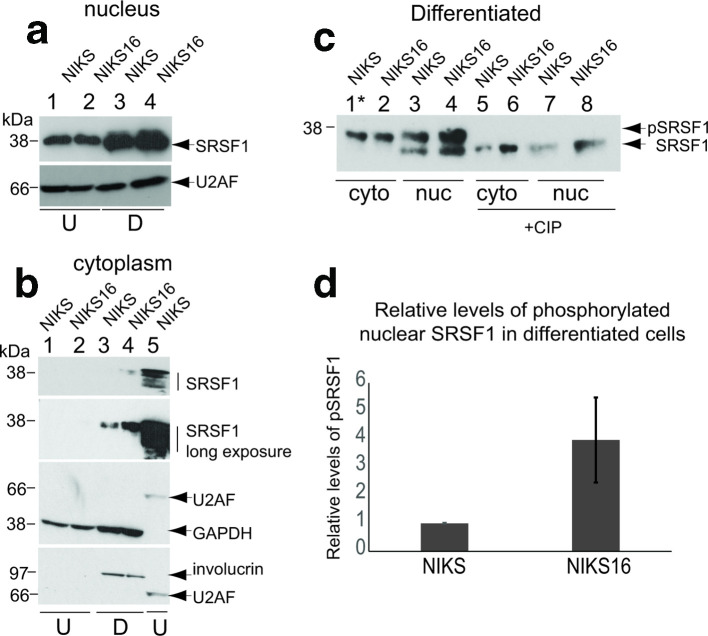
SRSF1 phosphorylation is upregulated by HPV16 infection in a differentiation-specific manner. (a) Western blot analysis of total SRSF1 (Mab96) levels in the nuclei of undifferentiated (U) and differentiated (D) NIKS (HPV-negative) and NIKS16 (HPV16-positive) keratinocytes. Nuclear U2AF^65^ was used as a loading control. (b) Hyper- and hypophosphorylated SRSF1 levels (Mab96 reactivity indicated with vertical lines) in the cytoplasm of undifferentiated (U, lanes 1 and 2) and differentiated (D, lanes 3 and 4) NIKS (HPV-negative) and NIKS16 (HPV16-positive) keratinocytes. Lane 5 shows nuclear SRSF1 from undifferentiated NIKS16 cells. Short (top panel) and long (second top panel) exposures of the western blot are shown to visualize cytoplasmic phosphorylated SRSF1. GAPDH was used as a cytoplasmic loading control. U2AF^65^ was used as a nuclear loading control. Involucrin staining showed that NIKS and NIKS16 cells were differentiated (lanes 3 and 4). Nuclear and cytoplasmic fractions were prepared from the same cells. Involucrin is a cytoplasmic protein so acts also as a differentiation control for the western blots in (a) (lanes 3 and 4). (c) Hyper- and hypophosphorylated SRSF1 levels (Mab96) in the nuclei (nuc) and cytoplasm (cyto) of differentiated NIKS (HPV-negative) and NIKS16 (HPV16-positive) keratinocytes. Lane 1, asterisk: five times the quantity of protein extract was applied to this lane. +CIP, protein extracts were digested with calf intestinal alkaline phosphatase to show the migration of hypophosphorylated SRSF1. (d) Quantification of the relative levels of phosphorylated nuclear SRSF1 in NIKS compared to NIKS16 cells. The data show the mean and sd from three separate experiments.

The nuclear location of SRSF proteins is positively controlled by phosphorylation of their serine–arginine (RS) domains, but cycles of phosphosphorylation/dephosphorylation are also important for SRSF function [13]. We reported previously that differentiated HPV-positive keratinocytes expressed more hyperphosphorylated SRSF1 than undifferentiated keratinocytes [36]. Using Mab96, which detects all forms of SRSF1, the protein was detected in the nuclear fractions from both NIKS and NIKS16 cells, but more SRSF1 was detected in differentiated (D) cells (Fig. 2a, lanes 3 and 4). U2AF was used as a nuclear splicing factor loading control. There was very little phosphorylated SRSF1 detected in the cytoplasm of HPV-negative or positive undifferentiated (U) NIKS cells (Fig. 2b, lanes 1 and 2). Upon long exposure of the western blot some SRSF1 was detected in the cytoplasm of differentiated (D) NIKS cells (Fig. 2b, lane 3). However, cytoplasmic SRSF1 levels were markedly increased in differentiated (D) NIKS16 cells (Fig. 2b, lane 4). The increased, cytoplasmic SRSF1 was phosphorylated because it had very similar mobility in the western blotted gel to hyperphosphorylated nuclear SRSF1 (Fig. 2B, lane 5). GAPDH was used as a loading control for cytoplasmic fractions (Fig. 2b, lanes 1–4). U2AF was used as a loading control for the nuclear fraction in lane 5. Involucrin antibody reactivity demonstrated differentiation of the protein extracts in lanes 3 and 4. To confirm that SRSF1 was phosphorylated, we carried out dephosphorylation of differentiated cell extracts using calf intestinal alkaline phosphatase (+CIP) and used a gradient gel that was able to separate SRSF1 isoforms. In order to visualize cytoplasmic SRSF1 from NIKS cells, five times the amount of protein lysate was loaded in lane 1 of Fig. 2c (asterisk). In the cytoplasmic compartment of both NIKS and NIKS16 cells, only hyperphosphorylated SRSF1 was detected (Fig. 2c, compare lanes 1 and 5 and lanes 2 and 6). Hyper- and hypo-phosphorylated SRSF1 was detected in the nucleus of NIKS (Fig. 2c, compare lanes 3 and 7) and NIKS16 cells (Fig. 2c, compare lanes 4 and 8), which also contained more SRSF1 overall. Quantification of relative levels of phosphorylated nuclear SRSF1 from three separate experiments revealed a 3.5-fold increase in differentiated NIKS16 compared to NIKS cells (Fig. 2d). Taken together, these data suggest that HPV infection upregulates SRSF1 protein phosphorylation and increases cytoplasmic SRSF1 levels during the productive stages of the HPV life cycle in differentiated keratinocytes.

### SRPK1 levels are increased by HPV infection

**Fig. 3.**
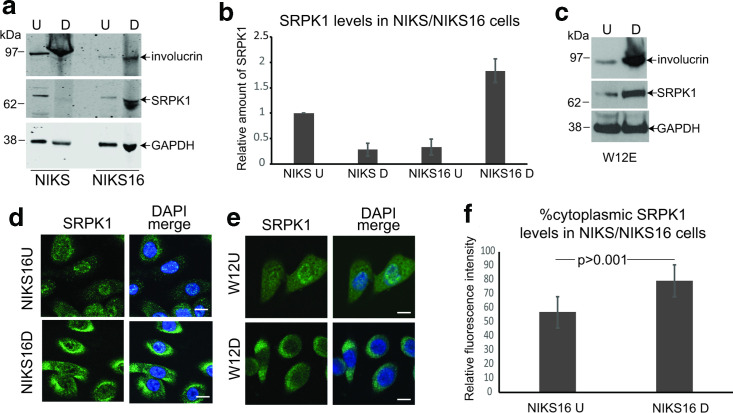
SRPK1 is greatest in differentiated HPV16-positive keratinocytes. (a) Western blot analysis of SRPK1 levels in undifferentiated (U) and differentiated (D) NIKS (HPV-negative) and NIKS16 cells (HPV16-positive) keratinocytes. (b) Graph showing quantification of SRPK1 levels relative to the GAPDH control. The mean and sd of three separate experiments are shown. (c) Western blot analysis of SRPK1 levels in undifferentiated (U) and differentiated (D) W12E (HPV16-positive) keratinocytes. In (a) and (c) involucrin was used to show keratinocyte differentiation and GAPDH was used as a loading control. (d) Confocal microscopy analysis of SRPK1 location in undifferentiated and differentiated NIKS16 keratinocytes. (e) Confocal microscopy analysis of SRPK1 location in undifferentiated (U) and differentiated (D) W12E (HPV16-positive) keratinocytes. DAPI merge, nuclei were counterstained with DAPI. Bar, 10 µm. (f) Graph showing quantification of cytoplasmic compared to total cellular SRPK1 levels in undifferentiated and differentiated NIKS16 cells. Twelve cells were measured in each case and the mean and sem are shown.

SRPK1 activity is required for nuclear SRSF import and function so we investigated whether, similar to its substrate SRSF proteins, SRPK1 was upregulated by HPV16 infection. Protein lysates were prepared from undifferentiated (U) and differentiated (D), HPV16-negative and -positive NIKS keratinocytes. Western blot analysis showed reduced levels of SRPK1 in differentiated compared to undifferentiated virus-negative cells (Fig. 3a, b, NIKS). In contrast, SRPK1 levels were increased in differentiated HPV16-positive NIKS cells (Fig. 3a, b, NIKS16). Similarly, compared to undifferentiated HPV16-infected W12E cells, levels of SRPK1 increased when the cells were differentiated (Fig. 3c). Increased involucrin levels indicated when NIKS, NIKS16 and W12E cells were differentiated (Fig. 3a, c). The cellular location of SRPK1 during the HPV16 life cycle was examined by confocal microscopy in HPV16-positive epithelial cells. In undifferentiated (U) and differentiated (D) NIKS16 cells, SRPK1 was located in both the nuclear and the cytoplasmic compartments, but in differentiated cells a greater proportion of SRPK1 was present in the cytoplasm (Fig. 3d). A very similar result was found for W12E cells (Fig. 3e). Quantification of levels of cytoplasmic SRPK1 in undifferentiated versus differentiated NIKS16 cells revealed a highly significant increase due to HPV16 infection (Fig. 3f). Thus, SRPK1 levels and subcellular location is controlled by HPV16 infection during keratinocyte differentiation.

### HPV E2 upregulates SRPK1

**Fig. 4.**
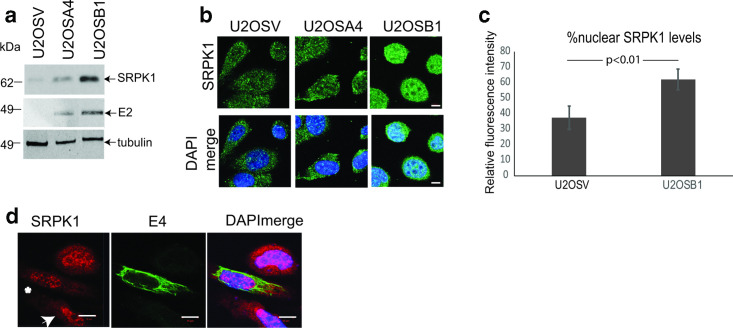
SRPK1 expression is regulated by HPV16 E2 but not by HPV16 E4. (a) Western blot analysis of levels of SRPK1 in U2OS cells stably transfected with an empty vector (U2OSV), or in two different U2OS cell clones stably transfected with an E2 expression vector (U2OSA4, U2OSB1). Tubulin was used as a loading control. (b) Confocal microscopy analysis of SRPK1 location in the three U2OS cell clones. Bar, 20 µm. (c) Graph showing quantification of nuclear as a percentage of total cellular SRPK1 levels in U2OSV and U2OSB1 cell clones. Five cells were measured in each case and the mean and sem are shown. (d) Confocal microscopy analysis of SRPK1 (red staining) location in HeLa cells overexpressing E4 (green staining, white asterisk) compared to untransfected cells (white arrowhead). DAPI merge, nucleic were stained with DAPI (blue). Bar, 10 µm.

Late viral regulatory proteins E2 and E4 have been shown to have an association with SRSF proteins and SRPK1: HPV16 E4 can bind SRPK1, which suggests E4 could be in complex with SR proteins, while the viral replication/transcription factor E2 has SRSF protein-like functions and activates expression of SRSF1, 2 and 3 [29, 44–46]. Therefore, we investigated if either E2 or E4 was responsible for the HPV-associated changes we observed in SRPK1 levels or subcellular location. First, we tested if E2 could increase SRPK1 levels. We compared SRPK1 levels in U2OS cells stably transfected with vector alone (U2OSV) and in two E2-positive U2OS clones, U2OSA4 and U2OSB1, which express different levels of HPV16 E2 (Fig. 4a) [35, 36]. SRPK1 expression responded to increased levels of E2 and was markedly upregulated in the B1 clone with the highest level of E2 expression (Fig. 4a). Confocal microscopy analysis of SRPK1 confirmed upregulation in U2OSB1 cells (Fig. 4b). Overexpression of E2 can drive cells into senescence or apoptosis [47]. Although U2OS cells are relatively resistant to these processes, the U2OSB1 cells appeared more rounded than the low E2-expressing cells (U2OSA4) or the E2-negative control cells (U2OSV), probably due to E2 toxicity, and this may influence SRPK1 location. Quantification of SRPK1 levels in the nucleus compared to the entire cell confirmed that increased SRPK1 was present in the nucleus of cells expressing E2 (Fig. 4c). These data suggest that HPV16 regulates expression levels of SRPK1 through the viral transcription factor E2.

To investigate if HPV16 E4 controlled SRPK1, we overexpressed E4 in HeLa cervical epithelial cells (we were unable to obtain sufficient expression levels in HaCaT or NIKS cells). Confocal microscopy analysis revealed that there was no apparent change in the subcellular location or levels of SRPK1 between cells expressing or not expressing E4 [compare the two cells indicated with an asterisk (E4-positive) and an arrow (E4-negative) in Fig. 4d].

### SRPK1 is required to maintain levels of SRSF1 in differentiated HPV16-positive keratinocytes

**Fig. 5.**
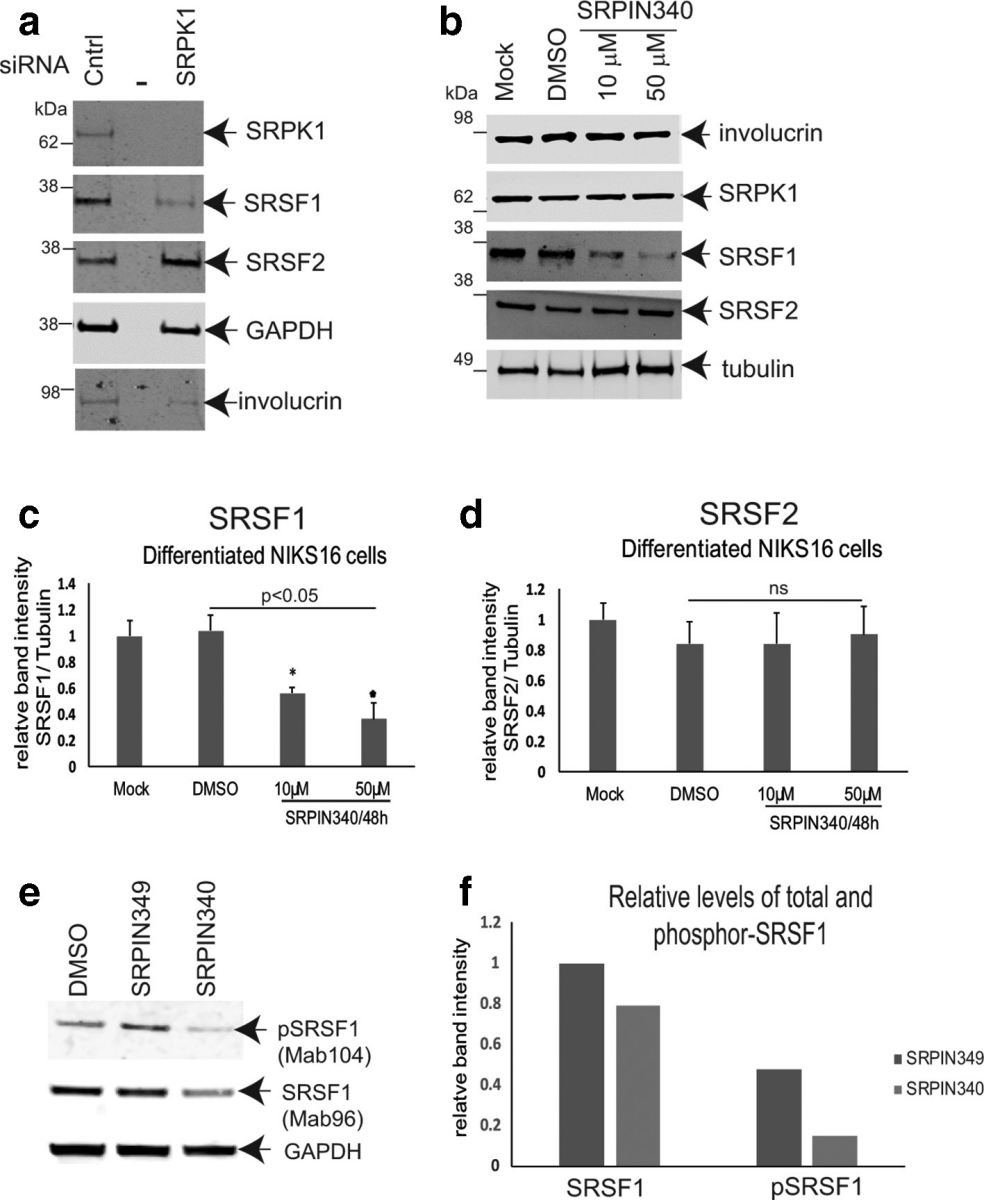
SRPK1 is required for SRSF1 phosphorylation in differentiated HPV16-positive keratinocytes. (a) Western blot analysis of total cellular SRSF1 levels (Mab96) in NIKS16 (HPV16-infected) cells treated with a control siRNA (Cntrl) or upon siRNA depletion of SRPK1 (SRPK1). The middle lane of the three is blank (–). GAPDH was used as a loading control. SRSF2 is shown as a control for an SR protein that is not significantly phosphorylated by SRPK1. Involucrin is shown as a control for keratinocyte differentiation. (b) Western blot analysis of total SRSF1 levels (Mab96) in NIKS16 cells untreated (Mock) or treated with the SRPK1 inhibitor SRPIN340 (10 µM, 50 µM) or with the vehicle, DMSO. SRPK1 levels are unaffected by drug treatment. SRSF2 is shown as a control for an SR protein that is not significantly phosphorylated by SRPK1. Involucrin is shown as a control for keratinocyte differentiation. Tubulin was used as a loading control. (c) Graph of relative levels of SRSF1 in untreated, DMSO-treated and SRPIN340-treated NIKS16 cells. (d) Graph of relative levels of SRSF2 in untreated, DMSO-treated and SRPIN340-treated NIKS16 cells. Western blot analysis of total (Mab96) and phosphor-SRSF1 (Mab104) levels in differentiated NIKS16 keratinocytes treated with drug vehicle, DMSO, or 10 µM SRPIN340 or SRPIN349, a similar compound that can bind SRPK1 but cannot inhibit its activity. (f) Quantification of the data in (e). The data are representative of two separate experiments.

HPV-mediated increases in SRPK1 levels could be responsible for causing the increased levels of phosphorylated SRSF1 that we observed in differentiated HPV-infected cells. siRNA depletion experiments revealed that loss of SRPK1 resulted in reduced total SRSF1 levels in differentiated NIKS16 cells. The effect on SRSF1 was specific because levels of another SR protein that is a much poorer substrate for SRPK1, SRSF2, did not change (Fig. 5a). Similarly, treatment of cells with the specific SRPK1 inhibitor SRPIN340 also caused a significant decrease in total SRSF1 levels (Fig. 5b, c), but there was no change in SRSF2 levels (Fig. 5b, d). Moreover, SRSF1 levels decreased in an SRPIN340 dose-dependent manner (Fig. 5c). To assess the effect of SRPIN340 on SRSF1 activity, we examined total SRSF1 (Mab96) and pSRSF1 (Mab104) levels in NIKS16 cells in the presence of the drug, or the drug vehicle, DMSO, or upon treatment with SRPIN349, a compound that can bind SRPK1 but does not inhibit its kinase activity [48]. Levels of total SRSF1 were again reduced in the presence of the kinase inhibitor, but there was a greater reduction in pSRSF1 compared to controls (Fig. 5e, f). This confirms that SRPK1 controls SRSF1 phosphorylation, and therefore its activity, in HPV16-infected keratinocytes.

These results suggest that SRPK1 is required to maintain levels of SRSF1 in differentiated HPV-infected keratinocytes. Taken together, our data reveal an HPV16-regulation of the SRPK1–SR protein control axis.

## DISCUSSION

We reported previously the upregulation of SRSF1 in differentiated W12 cells and in the upper epithelial layers of low-grade cervical lesions [36, 39]. Moreover, we showed that HPV16 and 31 E2 transcription factor was able to trans-activate the promoter of the gene encoding SRSF1 [30, 40]. Here we show that SRSF1 has increased cytoplasmic abundance in differentiated HPV16-infected keratinocytes. The change in location of a portion of the protein may be related to HPV infection because more cytoplasmic SRSF1 was detected in NIKS16 compared to HPV-negative NIKS cells. Nuclear and cytoplasmic levels of hyperphosphorylated SRSF1 were increased in differentiated HPV16-positive NIKS cells compared to HPV-negative NIKS cells. Although analysis of the relative levels of SRSF1 in both cellular compartments showed that the majority of the protein was located in the nucleus, the presence of increased hyperphosphorylated cytoplasmic SRSF1 suggests that the SRSF nuclear import machinery may be saturated by the increased amounts of the protein present in differentiated HPV-infected keratinocytes. Alternatively, late in the virus life cycle SRSF1 could be actively retained in the cytoplasm where it might have a specific cytoplasmic function, for example control of mRNA stability or translation.

SRPKs transduce growth signals to positively or negatively control SR protein phosphorylation and splicing [13]. SR proteins are imported into the nucleus following SRPK1-mediated phosphorylation, while cycles of phosphorylation/dephosphorylation regulate the various functions of SR proteins [13]. We have demonstrated that HPV16 upregulates the SR protein kinase, SRPK1, in a keratinocyte differentiation stage-specific manner. While SRPK1 levels were reduced upon differentiation of HPV16-negative keratinocytes, levels of the kinase were two-fold greater in differentiated HPV-infected cells compared to undifferentiated cells. Both nuclear and cytoplasmic SRPK1 were detected in HPV-infected keratinocytes. However, cytoplasmic levels of SRPK1 were increased in differentiated W12E cells and NIKS16 cells, compared to undifferentiated cells. This suggests that HPV infection can cause changes in the levels and subcellular distribution of SRPK1 similar to those of its substrate SRSF1.

HPV replication in the differentiating epithelium is subject to control by metabolic signalling alterations. For example, the Akt signalling pathway is activated by HPV E7 [49, 50], which is expressed during the early phase of infection in the lower layers of the epithelium [51]. Akt induces autophosphorylation of SRPK1 to allow its translocation to the nucleus where it upregulates SR protein phosphorylation and splicing in concert with a nuclear SR protein kinase, Clk [52]. This suggests that lower levels of Akt could result in a block to SRPK1 import into the nucleus. Expression of E7 is reduced in more differentiated HPV-infected keratinocytes, and this may explain the apparent increase in cytoplasmic SRPK1 levels in differentiated HPV16-infected cells. However, this could also be due to SRPK1 upregulation by HPV16 E2, which is expressed in keratinocytes in the mid- to upper epithelial layers [53]. E2 can regulate many cellular proteins at the transcriptional level [54] but direct E2 stimulation of SRPK1 expression remains to be explored. Analysis of the SRPK1 promoter sequence revealed a sequence **ACCG** TTGAAA **CGGT**. The nucleotides in bold conform to the consensus alpha E2 binding site sequence. However, although the nucleotide sequence of the central part of the E2 binding motif (underlined) can be variable, it is too long by 2 nt. Further experiments are required to see if E2 can bind directly to the SRPK1 promoter. Alternatively, it could bind indirectly via transcription factor partners such as SP1, which is known to activate SRPK1 expression [55].

Increased levels of SRSFs and SRPK1 have been detected in tumour cells [13]. Although the mechanisms behind this are unclear, it has been postulated that increased levels of splicing factors result in altered alternative splicing to create a tumorigenic transcriptome and/or by promoting genome instability [21]. One explanation for the increase in SRPK1 levels in HPV-infected cells could be that these cells represent tumour progression. We consider that this is unlikely because we could detect markers of keratinocyte differentiation in differentiated W12 and NIKS16 cells, which are not usually detected in tumour cells [56]. Moreover, these cells formed discrete colonies in monolayer culture and were able to form differentiated tissues in 3D organotypic raft cultures (data not shown). On the other hand, W12 cells were isolated from a low-grade cervical lesion [38], while clone 2L NIKS16 cells form a CIN1-like lesion when grown in 3D raft culture [34]. Therefore, the presence of these factors in the cytoplasm of HPV16-positive cells could be an early event in tumour progression.

Increased SRPK1 levels could be beneficial to viral replication. Activation of the SRPK1–SR protein axis late in HPV infection could be important for correct expression of alternatively spliced late mRNAs that encode the virus capsid proteins [4, 6]. To test the role of SRPK1 in regulating SRSF1 during the HPV16 life cycle we depleted levels of the kinase in differentiated NIKS16 keratinocytes using siRNA and also inhibited the kinase with SRPIN340, a specific inhibitor of SRPK1 [48]. Both treatments reduced the levels of total SRSF1 but drug treatment also reduced phospho-SRSF1 protein levels. A previous study in colorectal cancer cells revealed that reducing SRPK1 levels by drug or siRNA treatment caused SRSF1 to be degraded in the cytoplasm [57]. SRSF1 mRNA levels were unaltered upon siRNA or drug inhibition of SRPK1 in HPV-infected keratinocytes (data not shown), suggesting that SRSF1 may undergo proteasomal degradation in the cytoplasm of SRPK1-depleted, or inhibited, non-tumour keratinocytes. Therefore, SRSF1 phosphorylation in the cytoplasm, driven by HPV16 infection, may aid stabilization of the protein and its accumulation in that cellular compartment.

HPV gene expression is positively and negatively controlled largely at various post-transcriptional levels, particularly through control of viral pre-mRNA splicing [3, 4]. Because SR splicing factors are required for generation of HPV mRNAs, and SRPK1 is required to regulate these proteins, HPV gene regulation could be achieved by viral stimulation of these factors. The HPV life cycle is tightly linked to keratinocyte differentiation, so the differentiation-specific upregulation of SR proteins and their kinase that we have uncovered strengthens this hypothesis. Possible HPV regulation of Clk, the nuclear SR protein kinase and partner protein of SRPK1, and the role of Clk in the HPV life cycle, remain to be explored. It is of note that viral stimulation of SRPK1 activity could affect the HPV life cycle in other ways such as phosphorylation of viral proteins including E2 [28], which we have shown upregulates SRPK1 expression, suggesting a feedback mechanism. SRPK1 can phosphorylate HBV core protein, a prerequisite for encapisdation of viral DNA [24]. HPV capsid proteins, which are phosphorylated, are synthesized in differentiated keratinocytes. A recent study showed capsid protein phosphorylation was essential for virus entry, although the kinase has not been identified [58]. It is tempting to speculate that SRPK1 could be a candidate kinase for this event. If SRPK1 proves to be important for HPV replication, anti-splicing drugs such as SRPIN340 could be developed as novel antivirals against HPV.
